# Impact of Timing following Acute Myocardial Infarction on Efficacy and Safety of Bone Marrow Stem Cells Therapy: A Network Meta-Analysis

**DOI:** 10.1155/2016/1031794

**Published:** 2015-12-13

**Authors:** Bei Liu, Chong-Yang Duan, Cheng-Feng Luo, Cai-Wen Ou, Zhi-Ye Wu, Jian-Wu Zhang, Xiao-Bin Ni, Ping-Yan Chen, Min-Sheng Chen

**Affiliations:** ^1^Department of Cardiology, Zhu Jiang Hospital, Southern Medical University, Guangzhou 510280, China; ^2^Department of Biostatistics, School of Public Health and Tropical Medicine, Southern Medical University, Guangzhou 510515, China; ^3^Department of Cardiology, The Second Affiliated Hospital of Guangzhou Medical University, Guangzhou 510280, China; ^4^Southern Medical University, Guangzhou 510515, China

## Abstract

*Background*. The optimal timing for Bone Marrow Stem Cells (BMCs) therapy following acute myocardial infarction (AMI) remains unclear. *Aims*. To synthesize the evidence from trials using a multiple-treatment comparison method, thereby permitting a broader comparison across multiple timing of BMCs therapy. *Methods and Results*. Randomized controlled trials in patients with AMI receiving BMCs therapy were identified from PubMed, Ovid LWW, BIOSIS Previews, and the Cochrane Library through January 2015. 2 035 patients of 31 studies included in our analysis were allocated to 5 groups' treatments: 1~3 days, 4~7 days, 8~14 days, 15~30 days, or placebo/control group. The multiple-treatment meta-analysis showed that 4~7 days' group could lead to significantly increased left ventricular ejection fraction (LVEF) as compared with control (mean of MDs and 95% CI: 6 months, 3.05 (0.92~5.25); 12 months, 4.18 (2.30~5.84)). Only 4~7 days led to significant reduction of MACEs compared with control (OR and 95% CI 0.34 (0.13~0.96)) for 12-months follow-up. In simulated comparisons, the 4~7 days' group ranked better than other timing groups for improvement of LVEF or reduction of the incidence of major adverse cardiac events. *Conclusions*. 4~7 days after AMI might be the optimal timing for cell therapy in terms of efficacy or safety.

## 1. Introduction

In the past 15 years, numerous clinical trials on Bone Marrow Stem Cells (BMCs) treatment of acute myocardial infarction (AMI) have been carried out. Although the results were inconsistent, evidence from several meta-analyses demonstrated that BMCs treatment [1–3], as a kind of hopeful supplemental treatment beyond medication, percutaneous coronary intervention (PCI), and coronary artery bridge graft (CABG), could lead to moderate improvements in left ventricular (LV) function after AMI. Nevertheless, many fundamental questions such as optimal timing of BMCs delivery remain undefined [4].

After an AMI, the initial inflammatory myocardial milieu progressively changes to that of a remodeled heart [5]. Understandably, the timing of cell therapy in AMI patients may be a predominant factor affecting the result of injecting BMCs [6]. Based on this, several studies to date have focused on this issue. REPAIR-AMI trial showed that delivery of BMCs 5–7 days after AMI resulted in greater improvement in LV ejection fraction (LVEF) compared to earlier delivery [7]. Recently, specializing in the timing of BMC transplantation in AMI patients, TIME (Timing In Myocardial infarction Evaluation) [8] and LateTIME [9] trials which are applied within 7 days and 2-3 weeks, respectively, after AMI, unfortunately, failed to verify the beneficial effects of BMCs administration on either global or regional LV functions. Moreover, inconsistent results from several meta-analyses were also reported. In the largest meta-analysis [1], the reduction of LVEDV was significantly greater when BMCs were injected within 7 days after acute MI, while other outcomes were similar with BMCs injection during the 7–30-day interval after MI. However, another meta-analysis of acute MI trials reported a greater improvement in LVEF with BMC injection more than 7 days after AMI [3]. Thus, the optimal timing of cell therapy after AMI remains uncertain so that further exploration in this field is still necessary.

Based on previous published data, we report an overview of randomized controlled trials (RCTs) by means of multiple-treatment meta-analysis [10], also known as mixed-treatment comparisons meta-analysis or network meta-analysis which allows the integration of data from direct and indirect comparisons. We aimed to provide a useful summary of the results of the multiple-treatment meta-analysis that can be used to guide further study to apply cell therapy at a right timing after AMI.

## 2. Methods

### 2.1. Eligibility Criteria

Studies were included if they (1) were RCTs; (2) transplanted stem cells deriving from bone marrow with no restrictions in terms of dose, type, or administration route; (3) were conducted in patients with AMI; (4) were conducted in patients who received reperfusion therapy with no restrictions in terms of PCI or thrombolysis or CABG; and (5) involved participants in the comparator arm receiving standard therapy rather than stem/progenitor cells. The exclusion criteria for studies were as follows: (1) subjects with angina or chronic IHD were included; (2) trial design was not randomized; (3) no LVEF data were available; (4) transplanted cells were not derived from bone marrow, or circulating/peripheral progenitor cells were mobilized from bone marrow with granulocyte colony stimulating factor (G-CSF); (5) there was lack of control group; (6) BMCs were allogenic; or (7) trial reported timing of cell therapy which failed to fall into our defined timing group.

### 2.2. Study Selection and Data Collection

PubMed, Ovid LWW, BIOSIS Previews, and the Cochrane Library were searched through January 2015 using the following terms: “bone marrow,” “bone marrow cells,” “stem cells,” “stem cell,” “progenitor cell,” “progenitor cells,” “coronary artery disease,” “myocardial infarction,” “acute myocardial infarction,” “ischemic heart disease,” “ischemic cardiomyopathy,” “cardiomyopathy,” “cardiac repair,” and “heart failure.” In addition, we manually searched the reference lists of all original articles and previous systematic reviews.

Three persons within the reviewing team independently reviewed references and abstracts retrieved from the search, assessed the completeness of data abstraction, and confirmed quality rating. Relevant data regarding baseline characteristics, stem cell type, duration of follow-up, LVEF and LV volumes, and major adverse cardiac events (MACEs) which were defined as combination of all-cause mortality, heart failure, stent thrombosis, in-stent restenosis, target vessel revascularization, cerebrovascular event, and ventricular arrhythmia were extracted (as available) from individual studies. Magnetic resonance imaging (MRI) and single photon emission computed tomography (SPECT) data were preferred over echocardiographic data for primary analysis, where available. For studies with 2 or more intervention arms that involved different doses or different type of BMCs or different administering timing after AMI, each study was considered as conduction of two or more comparisons accordingly. Short term (3–6-month) and long term (12–24-month) outcome data were separately analyzed for each treatment approach.

### 2.3. Quality Assessment

The criteria established by Jüni et al. were used to assess the quality of included RCTs [11]. A quality rating of adequate, unclear, or inadequate was given to each study, according to the adequacy of the random allocation concealment and blinding, respectively.

### 2.4. Method Classification of the Timing of BMC Delivery

According to the method classification reported in the largest meta-analysis [1] and review from Bartunek et al. [4], the timing of BMC delivery was classified using two different methods as shown in Table 1. All subjects involved in included studies were initially divided into 4 groups: A group (1~7 days), B group (8~14 days), C group (15~30 days), and control group, respectively, based on different timing after AMI of BMCs delivery. Because the degree of inflammation in myocardial environment differs between the onset and last days of the first week after AMI, which might influence the effects of cell therapy, trials included in A group were further divided into A1 group (1~3 days) and A2 group (4~7 days) [4].

### 2.5. Outcome Measures

The primary endpoint was taken as changes in LVEF from baseline to follow-up. If the changes in LVEF cannot be obtained directly, we calculate the changes from the baseline LVEF and the follow-up LVEF by assuming that the correlation was 0.5. Changes in the LV end-diastolic volume (LVEDV), LV end-systolic volume (LVESV), and the incidence of MACEs were considered the secondary endpoints. The missing values of LVEDV and LVESV were imputed using the same way as LVEF if the necessary information can be obtained. All endpoints in 6-month follow-up and 12-month follow-up were compared separately.

### 2.6. Statistical Analysis

Both pairwise meta-analysis and multiple-treatment meta-analysis were performed in comparing the efficiency and safety of BMCs therapy. The relative effect sizes were calculated as mean differences (MDs) for the changes in LVEF and standardized mean differences (Hedges' *g*) were used for the changes in the LVEDV and LVESV. For binary outcomes, odds ratios (ORs) were used. Pairwise meta-analysis was carried out by synthesizing studies in order to make comparison among different timing groups and the controls. The *Q* test and *I* ^2^ were used to evaluate statistical heterogeneity. *I* ^2^ values of 25%, 50%, and 75% were considered evidence of low, moderate, and severe statistical heterogeneity, respectively. If *I* ^2^ > 50% or *Q* test *P* value <0.1, we considered it heterogeneous and a random effects model was used; otherwise, a fixed effect model was used.

Multiple-treatment meta-analysis combines direct and indirect evidence for all relative treatment effects and provides estimates with maximum power. The model was fitted into a Bayesian context with hierarchical models. A common heterogeneity parameter was assumed for all comparisons. All types of effect sizes are reported with their 95% credible intervals (CIs). In our study we only used the model with assumed consistency, for almost all the evidence used for the comparison of different timing of BMC delivery in the network was indirect. Sensitivity analyses were conducted on the primary outcome to test its robustness. A funnel-plot and Egger's test were applied to explore small-study effects. All tests were two-tailed and *P* < 0.05 was considered as significant in the meta-analysis. All data analyses were performed using the *R* statistical software (version 3.1.2). The “meta” package (version 4.1-0) and “gemtc” package (version 0.6-1) were used to perform the meta-analysis.

## 3. Results

### 3.1. Search Results

The search identified 731 unique publications which were screened. After excluding 609 publications based on title/abstract, full-text analysis was performed on 122 reports. Finally, 31 articles meeting our including criteria could be included in our analysis (Figure S1 in Supplementary Material available online at http://dx.doi.org/10.1155/2016/1031794).

### 3.2. Study Characteristics

A total of 2035 subjects were included and randomly assigned to BMCs group (*n* = 1025) and standard therapy (*n* = 830). Characteristics of the studies included in this analysis were shown in Table S1. Of the included studies, 11 reported data of both short and long term follow-up [12–27]. One study only reported data of 12-month follow-up [28]. 18 studies reported data ranging from 3 to 6 months of follow-up [8, 9, 29–44]. There were 4 multiple-arm studies which were divided into independent comparisons in terms of different cell dosage [14], timing [8, 31], and type of stem cell [40]. For outcome analysis, 31 comparisons from 30 studies could be available for LVEF based on data of 6-month follow-up and 13 comparisons based on 12-month follow-up. 20 comparisons from 19 studies for MACEs could be available based on data of 6-month follow-up and 14 comparisons based on 12-month follow-up. 26 comparisons for LVESV from 25 studies and 28 for LVEDV from 27 studies could be carried out, respectively, in our analysis. Funnel plots (Figure S2) and Egger's tests (*P* = 0.810 for LVEF, *P* = 0.663 for MACEs, resp.) showed little evidence of publication bias.

### 3.3. Study Quality

The quality metrics of included RCTs are shown in Table S2. At least 14 RCTs failed to blind participants and/or caregivers. There was insufficient information on blinding of participants and caregivers from 6 RCTs. The description on blinding of outcome was unclear in 3 RCTs. The follow-up was complete in most studies with shorter follow-up duration. In studies with longer follow-up, the percent of patients lost to follow-up was acceptable. The interreviewer agreement on these quality domains was greater than 90%.

### 3.4. Cardiac Parameters

**Figure 1:**
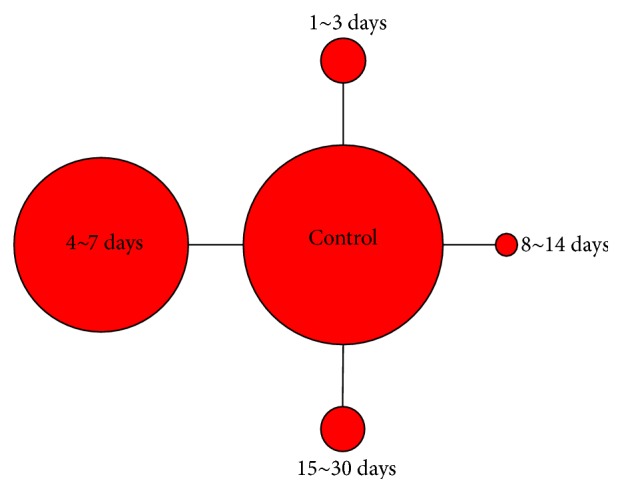
Network of treatment comparisons for overall efficacy in terms of ventricular function. The size of the nodes corresponds to the number of randomised participants (sample size). Directly comparable treatments are linked with a line, the thickness of which corresponds to the number of trials that assess the comparison.

**Figure 2:**
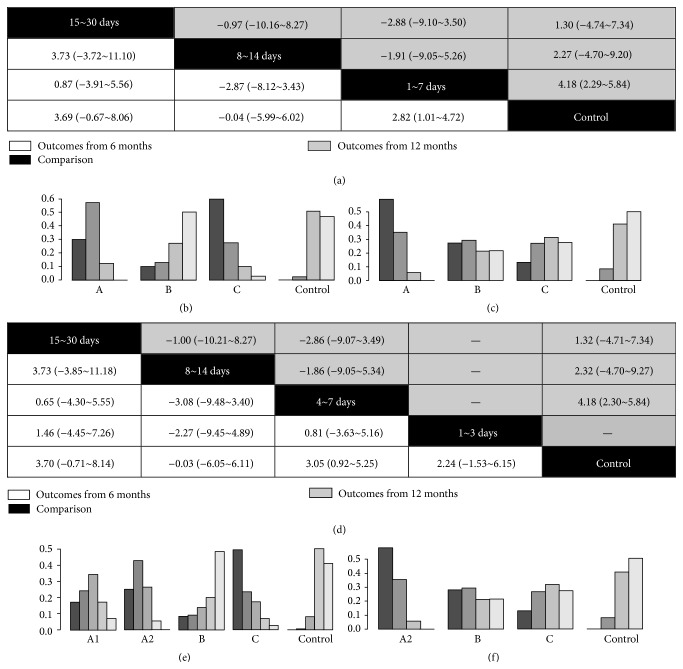
The multiple comparisons of stem cells therapy among different timing groups for left ventricular ejection fraction. (a) The multiple-treatment meta-analysis of left ventricular ejection fraction based on data of 6-month and 12-month follow-up according to grouping method 1. (b) The hierarchical rank of timing group for left ventricular ejection fraction based on data of 6-month follow-up according to grouping method 1. (c) The hierarchical rank of timing group for left ventricular ejection fraction based on data of 12-month follow-up according to grouping method 1. (d) The multiple-treatment meta-analysis of left ventricular ejection fraction based on data of 6-month and 12-month follow-up according to grouping method 2. (e) The hierarchical rank of timing group for left ventricular ejection fraction based on data of 6-month follow-up according to grouping method 2. (f) The hierarchical rank of timing group for left ventricular ejection fraction based on data of 12-month follow-up according to grouping method 2.

**Figure 3:**
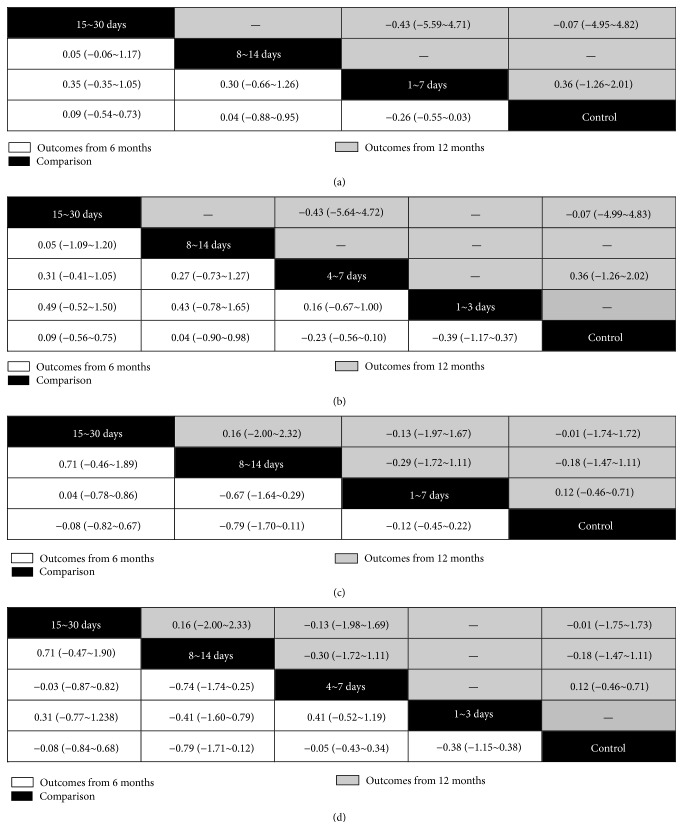
The multiple comparisons of stem cells therapy among different timing groups for myocardial remodeling. (a) The multiple-treatment meta-analysis of left ventricular end-systolic volume based on data of 6-month and 12-month follow-up according to grouping method 1. (b) The multiple-treatment meta-analysis of left ventricular end-systolic volume based on data of 6-month and 12-month follow-up according to grouping method 2. (c) The multiple-treatment meta-analysis of left ventricular end-diastolic volume based on data of 6-month and 12-month follow-up according to grouping method 1. (d) The multiple-treatment meta-analysis of left ventricular end-systolic volume based on data of 6-month and 12-month follow-up according to grouping method 2.

The networks of eligible comparisons are shown in Figure 1. The results of pairwise meta-analysis showed that the BMCs therapy resulted in a significant increase of LVEF compared with standard therapy (MD and 95% CI: 6 months, 2.53 (1.25~3.82); 12 months 4.09 (2.83~5.34)) (Table 2). The subgroup analysis showed that only the 4~7 days' group experienced significantly greater improvement in LVEF (MD and 95% CI: 2.85 (1.61~4.09) for 6 months; 4.34 (2.98~5.69) for 12 months). Results of the multiple-treatment meta-analysis of LVEF showed that 1~7 days' group significantly increased LVEF compared with control group regardless of duration of follow-up (Figure 2(a)). Interestingly, after further dividing 1~7 days' group into 1~3 and 4~7 days' group, the former only showed a trend toward improved LVEF, but the latter still resulted in a 3.05% (95% CI, 0.92~5.25) increase for 6-month follow-up and 4.18% (95% CI, 2.30~5.84) increase for 12-month follow-up in LVEF, respectively (Figure 2(d)). Trends toward increased LVEF in 4~7 days' group as compared with other timing groups can be observed. We created hierarchies of effect size for change of LVEF showed the distribution of probabilities of each group being ranked at each of the possible positions. For the outcome of LVEF, 15~30 days' group was one of the most best-performing groups in 6-month follow-up (Figures 2(b) and 2(e)) but not in 12-month follow-up (Figures 2(c) and 2(f)). The 4~7 days' group was the most efficacious group among all timing groups for results from both the 6-month and 12-month follow-up (Figures 2(e) and 2(f)). In terms of LVESV and LVEDV, no statistical differences could be observed among all groups (Figure 3).

### 3.5. Impact of BMC Therapy on Survival and Clinical Outcomes

**Figure 4:**
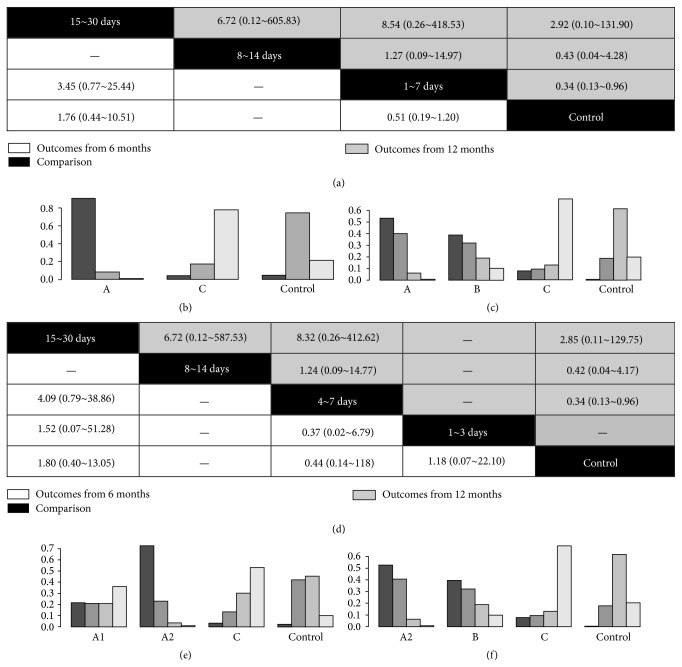
The multiple comparisons of stem cells therapy among different timing groups for major adverse cardiac events. (a) The multiple-treatment meta-analysis of major adverse cardiac events based on data of 6-month and 12-month follow-up according to grouping method 1. (b) The hierarchical rank of timing group for the incidence of major adverse cardiac events based on data of 6-month follow-up according to grouping method 1. (c) The hierarchical rank of timing group for the incidence of major adverse cardiac events based on data of 12-month follow-up according to grouping method 1. (d) The multiple-treatment meta-analysis of major adverse cardiac events based on data of 6-month and 12-month follow-up according to grouping method 2. (e) The hierarchical rank of timing group for the incidence of major adverse cardiac events based on data of 6-month follow-up according to grouping method 2. (f) The hierarchical rank of timing group for the incidence of major adverse cardiac events based on data of 12-month follow-up according to grouping method 2.

For the incidence of MACEs, results of pairwise meta-analysis (Table 2) from 12-month follow-up showed a statistical reduction of MACEs in patients with BMCs treatment compared with that in those with standard therapy (ORs and 95% CI: 0.45 (0.27~0.76)), but the results from 6-month follow-up only revealed a trend toward reduced MACEs (ORs and 95% CI: 0.66 (0.43~1.01)). The subgroup analysis showed that only subjects in 4~7 days' group experienced less MACEs compared with those who in control group based on comparison from both 6-month (OR and 95% CI: 0.44 (0.26~0.76)) and 12-month follow-up (OR and 95% CI: 0.31 (0.18~0.55)). The results of the multiple-treatment meta-analysis (Figure 4) showed that all comparisons of MACEs were not significant. Results from hierarchies of MACEs demonstrated that the 4~7 days' group also had the least incidence of MACEs compared with other groups regardless of duration of follow-up (Figures 4(e) and 4(f)).

## 4. Discussion

Our findings based on 31 RCTs involving 2035 subjects can be summarized as follows briefly: (1) only patients receiving BMCs during 4~7 days after AMI had a statistically significant improvement of LVEF regardless of duration of follow-up; (2) accordingly, the least incidence of MACEs can also be observed in patients receiving BMCs during 4–7 days after AMI.

The potential mechanisms for the optimal timing of BMCs transplantation after AMI are still under research. Shortly after acute MI, on the one hand, greater expression of chemoattractant [45] and adhesion molecules [4] in the acutely infracted heart may promote stem cells retention. On the other hand, the abundance of proinflammatory molecules may also cause excessive cell death [4, 8, 9]. In order to determine the optimal time for cell delivery after MI, how to strike a balance between these two opposite effects should be well clarified in the future.

### 4.1. BMCs Therapy in the First Week after AMI

Present results from our meta-analysis showed that BMCs delivered within the first week after MI were safe in terms of reduced MACEs and effective in terms of improved LVEF significantly. In order to narrow the frame of the optimal timing of BMCs treatment, studies applying BMCs therapy within 7 days after AMI were further divided into 1~3 days and 4~7 days after AMI. Interestingly, in patients with BMCs therapy within 3 days after AMI, results from 6 months of follow-up only showed a beneficial trend toward increased LVEF. Yet in patients with BMCs therapy during 4~7 days after AMI, both 6-month and 12-month results still maintain an improved LVEF statistically. The mechanisms behind these different results remain unclear. To date, only TIME trial tried to directly compare the effects of BMCs therapy between 3 days and 7 days as the cell injection timing after AMI [8]. Unfortunately, improved LVEF as primary endpoint was not observed in patients receiving BMCs therapy at these two timings.

In terms of trials supporting BMCs therapy within 3 days after AMI, the improved LV function might be due to the upregulated expression of cytoprotective proteins immediately (range from 1 to 4 days) after AMI, such as heat shock proteins 70 and 32 and blood vascular endothelial growth factor (VEGF) which was considered as a favorable factor of BMCs treatment [46]. For trials which failed to prove a positive relation between BMCs therapy and improved LV function [36–38], possible explanations cannot be ruled out in which the hostile microenvironment within the infarct tissue might have an adverse impact on BMCs survival, which might play a dominant role within 3 days after AMI due to a dramatic release of proinflammatory factor and ROS [47, 48]. Besides, that progressive increase of microvascular obstruction within the first 48 hours after reperfusion could impair inflow of oxygen and nutrients to support stem cell survival [49]. In order to investigate the pathophysiology of BMCs therapy in such setting of acute inflammation environment, more preclinicalstudies should be done at a cellular and molecular level.

Based on the existing data, the majority of trials have applied 4~7 days following AMI as BMCs delivery timing, accounting for about 66.7% of trials included in our meta-analysis. Consistent with both REPAIR-AMI trial [16–18] and BOOST trial [24, 25], our results indicated that BMCs therapy during 4–7 days after AMI resulted in greater improvement in LVEF compared with standard therapy. The mechanism for the above statistical benefits might come from the maximum degree of synergy of several well-confirmed mechanisms accounting for the cardiac repair of BMCs therapy during this time frame. Firstly, stem cells themselves can inhibit the inflammation. Following MI, myocardial necrosis initiates an inflammatory response that includes a cascade of cytokines and chemokines followed by recruitment of neutrophils and macrophages. The neutrophil (CD11b+/Ly6G+) infiltration increases immediately and endures to peak until days 1–3 and gradually decreases on day 5 [50]. Classical macrophages remain dominant up to day 5 displaying the classical M1 surface marker and functionally expressing higher levels of proinflammatory mediators. However, alternative macrophages' (M2) population remains predominant after day 5. Meanwhile, dendritic cells' accumulation reaches peak at days 5–7 while regulatory T cells, B cells, natural killer (NK) cells, and NKT cells increased gradually and peak at day 7 [51]. During this period following MI, stem cells therapy provides a rich source of cytokines and growth factors that regulate cell behavior. It has been shown that MSC treatment increases production of IL-6, subsequently preventing apoptosis by activated neutrophils through STAT3 transcription factors [52]. Besides, the increased production of IL-6 attenuates the respiratory burst, thereby regulating neutrophil activation. As a key event in myocardium repair, the macrophage polarization switch from M1 to M2 can be enhanced by MSC transplantations after MI; increased M2 macrophages further perform anti-inflammatory effects. Furthermore, T cell, natural killer cell, and B cell proliferation can also be suppressed by MSC paracrine factors. Relieved inflammation environment facilitates stem cell survival and prevents subsequent myocardial remodeling. Secondly, 48 hours following reperfusion, microvascular obstruction begins to be relieved and ROS decreases, which can also assist injected BMCs survival and engraftment. Thirdly, within hours of MI, there is a well-documented increase in circulating progenitor cells released from bone marrow that may contribute to myocardial repair [53]. A variety of cell surface markers can be detected in these stem cells, including CD34, c-kit, c-met, vascular endothelial growth factor- (VEGF-) 2R, and CXCR4 that actively participate in cardiac repair, in part through homing in response to gradients of VEGF, hepatocyte growth factor-1, and SDF-1 [40]. The peak release of these bone marrow-derived cells has been measured in around 7 days after AMI [54]. Thus, an underlying synergistic effect from injected and circulating progenitor cells might be involved in the process of cardiac repair. Finally, both substrate composition and stiffness can regulate cardiac differentiation potential of stem cells. In general, infarct extracellular matrix (ECM) stiffness increased slightly in the first week of AMI, but, during 2 and 4 weeks after AMI, the most dramatic alteration in stiffness occurred with a nearly threefold increase in stiffness. Moreover, previous study reported an increase in MMP expression within 2 days and maximal expression by 7 days after coronary artery ligation, which could explain decreases in ECM protein content. Results from Guo et al. showed that the early infarct matrix at 1 week after AMI failed to negatively influence the expression of either transcription factor [55] while the increased stiffness characteristic of the infarct later significantly abrogated the differentiation capacity of the cells. Thus, during first week following AMI, such a hypothesis might match reality more exactly—there exists an optimal extracellular environment which enables enhancing cellular differentiation and ultimately improves myocardial regeneration.

### 4.2. BMCs Therapy beyond 1 Week after AMI

In a real clinical practice, for some particular patients who might be too sick to receive BMCs therapy immediately or be treated initially in a hospital where BMCs therapy was not available after the onset of acute MI, naturally, delayed cell therapy may be taken as a consideration for clinical treatment [9]. Unfortunately, several RCTs applying 2-3 weeks as their timing of BMCs transplantation strategy failed to demonstrate improvement in LV function [9, 31, 36]. In agreement with our results, delivered BMCs beyond 7 days after AMI failed to reach a statistical improvement of LV function in terms or either LVEDV, LVESV, or LVEF. Considering the fact that the postinfarction myocardium experiences a time-dependent stiffness change from flexible to rigid due to myocardial remodeling following tissue necrosis and massive ECM deposition, we presume that the timing beyond 1 week after AMI may not be superior to first week in terms of BMCs therapy for AMI. Just like the comments above, scar forms progressively on the one hand in the infarcted region during this time so that it becomes more and more difficult for engraftment and interaction with the niche of the infused BMCs to achieve cardiac repair [27]. On the other hand, a possible synergistic effect between intracoronary delivered and circulating progenitor cells which reduced along with the decrease of chemotactic factors with time may not have become possible.

### 4.3. Safety

Interestingly, compatible with improved LVEF observed in 4~7 days group, subgroup analysis also revealed a significant reduction of MACEs in this subgroup population compared with patients without cell therapy regardless of duration of follow-up. Although results from multiple-treatment meta-analysis failed to reach significance as to comparisons among timing groups, at least, that could indicate the absence of increased adverse events, suggesting that BMSs therapy is safe.

### 4.4. Limitations

There are some limitations to our analysis that should be taken into account. Firstly, majority of studies included in our analysis applied the first week after AMI as the timing of cell therapy, while lesser ones applied other timings, which could lead to an uneven distribution of sample size. Secondly, due to maldistribution of studies among different timing groups, it was a pity that further analysis could not be done in the context of considering other underlying factors including cell dosage, cell type, cell isolation protocols, storage methods, and image modalities, which might be the sources of heterogeneity. Moreover, the selection of outcome variables might also be a source of limitation. LVEF as primary endpoint in current analysis has been known to be load dependent and be influenced by compensatory hypercontractile segments in the viable myocardium. Also, its prognostic significance is limited while with values >45%. Thus, it is necessary to identify a combination of parameters in order to reflect the true impact of BMCs therapy in AMI patients.

## 5. Conclusion

This is, to our knowledge, the first multitreatment meta-analysis targeted to assess the impacts of timing on efficacy and safety of BMCs therapy in AMI patients. A modest but significant improvement of LV function along with reduced MACEs can be observed when AMI patients received BMCs therapy during 4~7 days following acute ischemia, but not observed during other timings (1~3 days, 8~14 days, and 15~30 days). Thus, before the presence of more valid date, 4~7 days as timing of cell therapy in patients with AMI might be considered preferably when making a clinical trial design. Importantly, the underlying mechanisms behind impacts of timing should be explored in detail.

## Supplementary Material

731 citations were initially identified. After exclusion of 609 according to titles and abstracts, 122 RCTs were examined according to our defined selection criteria. Of which, 48 involving ineligible patients, 12 with non-randomized design, 13 without LVEF as endpoint, 9 utilizing stem cells beyond bone marrow derived ones, 5 lacking controls as comparison, 1 applying allogenic stem cells, and 3 applying timing of cell therapy failed to follow into our defined timing group were rejected. Finally, 31 RCTs were involved in present analysis.

## Figures and Tables

**Table 1:** Grouping method of timing of Bone Marrow Stem Cells therapy.

Method 1	Method 2
A: 1~7 days	A1: 1~3 days
	A2: 4~7 days

B: 8~14 days	B: 8~14 days

C: 15~30 days	C: 15~30 days

Control	Control

**Table 2:** Pairwise comparisons of efficacy or safety in stem cells-treated patients compared with patients receiving standard therapy.

Groups versus control^#^	LVEF				MACE			
	Number of studies	Number of patients	*I* ^2^	MD (95% CI)	Number of studies	Number of patients	*I* ^2^	OR (95% CI)
*6 months*								
1~7 days	23^*∗*^	862/673	71.8	2.57 (1.21~3.94)	16	768/571	0.0	0.51 (0.31~0.82)
1~3 days	6	142/127	36.2	1.26 (−0.66~3.18)	4	143/104	0.0	1.03 (0.30~3.47)
4~7 days	18	881/674	72.3	2.85 (1.61~4.09)	12	625/467	0.0	**0.44 (0.26~0.76)**
8~14 days	3	34/30	0.0	−0.84 (−4.31~2.64)	0	0	—	—
15~30 days	5	187/169	87.1	3.27 (−1.94~8.48)	4	167/140	0.0	1.79 (0.66~4.82)
Total	30	1083/872	73.6	2.53 (1.25~3.82)	19^$^	935/711	0.0	0.66 (0.43~1.01)
*12 months*								
1~7 days	10	311/240	50.0	4.34 (2.98~5.69)	9	342/296	12.7	0.31 (0.18~0.55)
1~3 days	—	—	—	—	—	—	—	—
4~7 days	10	311/240	50.0	4.34 (2.98~5.69)	9	342/296	12.7	**0.31 (0.18~0.55)**
8~14 days	2	10/10	0.0	2.33 (−3.95~8.62)	4	42/35	0.0	0.65 (0.15~2.84)
15~30 days	1	19/20	—	1.30 (−3.09~5.69)	1	21/21	—	2.11 (0.18~25.17)
Total	13	340/270	42.6	4.09 (2.83~5.34)	14	405/352	0.0	0.45 (0.27~0.76)

^*∗*^One study had arms belonging to 1~3 days' group and another one belonging to 8~14 days' group; ^$^one study had arms belonging to 8~14 days' group and another one belonging to 15~30 days' group. ^#^Multiarm studies were divided into independent studies.
