# Supplementary material for: Impact of Timing following Acute Myocardial Infarction on Efficacy and Safety of Bone Marrow Stem Cells Therapy: A Network Meta-Analysis
Source: Stem Cells Int. 2015 Dec 13;2016:1031794. doi: 10.1155/2016/1031794 (PMC4691493; doi:10.1155/2016/1031794)
Supplement: Supplementary file 1 — 731 citations were initially identified. After exclusion of 609 according to titles and abstracts, 122 RCTs were examined according to our defined selection criteria. Of which, 48 involving ineligible patients, 12 with non-randomized design, 13 without LVEF as endpoint, 9 utilizing stem cells beyond bone marrow derived ones, 5 lacking controls as comparison, 1 applying allogenic stem cells, and 3 applying timing of cell therapy failed to follow into our defined timing group were rejected. Finally, 31 RCTs were involved in present analysis. [file 1031794.f1.pdf]

**Supplementary Table S1. Characteristics of Studies Included in the Meta-Analysis**

| Source | N (SCs vs. control) | Age,yrs SCs | Age,yrs Control | Cell type | Number of injected cells($10^6$) | Route of injection | Time From AMI to injection (days) | Imaging modalities | Follow-up (months) |
|---|---|---|---|---|---|---|---|---|---|
| Assmus B (2010) | 204(1:1) | 56(11) | | BMCs | 236(174) | IC | 3-7# | Echo/ cMRI | 6,24 |
| Cao F (2009) | 86(1:1) | 50.7(1.1) | 51.0(1.0) | BMMNCs | 50(12) | IC | 7 | Echo/SPECT | 6,12 |
| Colombo A (2011) | 10(1:1) | 54(47-60)Δ | 56 (44–58)Δ | CD133+ cells | 5.9[4.9-13.5]Δ | IC | 10-14# | Echo/SPECT | 12 |
| Castellani M (2010) | 10(1:1) | 30-64# | | CD133+ cells | 5.9 (4.9 6-13.5) Δ | IC | 11-15# | Echo/SPECT/cMRI | 3,6,12 |
| Chen SL (2004) | 69(1:1) | 58(7) | 57(5) | MSCs | 48000-60000# | IC | 18.4(0.5) | Echo | 3,6 |
| Ge J (2006) | 20(1:1) | 58(11) | 59(8) | BMMNCs | 40 | IC | < 1 | Echo/SPECT | 6 |
| GrajeK S (2010) | 45(1:1) | 49.9 (8.4) | 50.9 (9.3) | BMCs | 410(180) | IC | 4-6# | Echo/SPECT/cMRI | 6,12 |
| Gao LR (2013) | 43(1:1) | 55.0 (1.6)* | 58.6 (2.5)* | MSCs | 3.08(0.52)* | IC | 17.1(0.6)* | Echo/SPECT | 6,12,24 |
| Hirsch A (2011) | 134(1:1) | 56(9) | 55(10) | BMMNCs | 296(164) | IC | 3-8# | Echo/cMRI | 4 |
| Herbots L (2009) | 67(1:1) | 55(11) | 58(10) | BMCs | 172(72) | IC | <1 | Echo/cMRI | 4 |
| Huang RC (2006) | 40(1:1) | 57.3 (10.1) | 56.7 (9.2) | BMCs | 420(180) | IC | <1 | Echo | 6 |
| Huikuri HV (2008) | 80(1:1) | 60(10) | 59(10) | BMMNCs | 360 | IC | 2-6# | Echo/ LV Angio | 6 |
| Janssens S (2006) | 67(1:1) | 55.8(11) | 57.9(10) | BMCs | 172(72) | IC | < 1 | Echo/cMRI | 4 |
| Lee JM (2014) | 58 (1:1) | 53.9 (10.5) | 54.2 (7.7) | MSCs | 72(9) | IC | 25(2.4) | Echo/SPECT | 6 |
| Meyer GP (2006) | 60(1:1) | 53.4(14.8) | 59.2(13.5) | BMCs | 2460(940) | IC | 4.8(1.3) | cMRI | 6,18 |
| Meluzín, J (2008) | 60(1:1:1) | 54 (2)* 54 (2)* | 55 (2)* | BMMNCs | 10 100 | IC | 5-9# | Echo/ SPECT | 6,12 |
| Nogueira FB （2009） | 20(2:1) | 59.7 (14.3) | 57.2 (10.8) | BMMNCs | 100 100 | IC | 5.5 (1.28) | Echo | 3,6 |
| Piepoli MF (2010) | 38(1:1) | 63.1 (2.4)* | 67.0 (2.7)* | BMMNCs | 418 | IC | 4-8# | Echo/ SPECT | 3,6,12 |

| | | | | | | | | |
|---|---|---|---|---|---|---|---|---|
| Plewka M (2011) | 56(2:1) | 56（9） | BMMNCs | 144（49） | IC | 7 | Echo | 6 |
| Penicka M (2007) | 27(2:1) | 59 (12) | BMMNCs | 2640(1960-3300)$^{\Delta}$ | IC | 9 (4-11)$^{\Delta}$ | Echo | 4 |
| Quyyumi AA (2011) | 31(1:1) | 52.0 (47.5-63.5)$^{\Delta}$ 52.0 (47.0-57.0)$^{\Delta}$ | CD34+ cells | 9.7（4.1） | IC | 8.3 | Echo/SPECT/cMRI | 3，6 |
| Suárezde Lezo J (2007) | 20(1:1) | 52(12) 55(11) | BMMNCs | 900(300) | IC | 7(5-12)$^{\Delta}$ | Echo | 3 |
| Sürder D (2013) | 195(1:1:1) | 55(15)$^{*}$ 62 (15)$^{*}$ 56 (14.5)$^{*}$ | BMMNCs | 153 153 | IC | 5 -7$^{\#}$ 21 -28$^{\#}$ | cMRI | 4 |
| Tendera M (2009) | 200(2:2:1) | 55 (37–74)$^{\Delta}$ 58 (30–75)$^{\Delta}$ 59 (37–73)$^{\Delta}$ | BMMNCs CD34+ CXCR4+ cells | 178 1.90 | IC | 3-12$^{\#}$ | cMRI | 6 |
| Traverse JH$^{\&}$ (2012) | 67(2:1) | 55.6 (10.8) 57.0 (12.4) | BMMNCs | 146(22.3) | IC | 3 | cMRI | 6 |
| Traverse JH$^{\&}$ (2012) | 53(2:1) | 58.2 (11.3) 57.0 (8.0) | BMMNCs | 146(12) | IC | 7 | cMRI | 6 |
| Traverse JH (2010) | 40(3:1) | 52.5 (43-64)$^{\Delta}$ 57.5 (54-59)$^{\Delta}$ | BMMNCs | 100 | IC | 3-10$^{\#}$ | Echo/cMRI | 3,6 |
| Traverse JH (2011) | 87(2:1) | 57（11） 54.6(11) | BMMNCs | 150 | IC | 14-21$^{\#}$ | Echo/cMRI | 6 |
| Turan RG (2012) | 62(2:1) | 61(15) 60(11) | BMMNCs | 96(32) | IC | 7 | Echo | 6,12 |
| Wöhrle J (2013) | 42(2:1) | 61.0（8.1） 61.1 (9.3) | BMMNCs | 381 | IC | 5-7$^{\#}$ | Echo/ cMRI | 6 |
| Wen R (2005) | 20(1:1) | 58(7) | BMCs | NR | IC | <1 | Echo | 6 |
| Yao K (2009) | 24(1:1) | 52.1 (6.3) 52.7 (7.8) | BMCs | 190(120) | IC | 3-7$^{\#}$ | Echo/ SPECT/cMRI | 6，12 |

Note: Values are presented as mean (SD) or mean. N: number of subjects. SC: stem cells. AMI: Acute Myocardial Infarction. BMCs: Bone Marrow Cells. IC: Intracoronary. Echo: Echocardiography. cMRI: Cardiac Magnetic Resonance Imagine. BMMNCs: Bone Marrow Mononuclear Cells. SPECT: Single-Photon Emission Computed Tomography. NR: Not Record. MSCs: Mesenchymal Stem Cells. LV Angio: Left Ventricular Angiography.$^{\Delta}$**:** Median (ranges). $^{\#}$: range. *: mean (SE). $^{\&}$: from same study

## Supplementary Table S2. Quality Assessment Scale for the Randomized Controlled Trials Included in the Meta-Analysis

| | Selection | | | Performance were | Detection | attrition | |
|---|---|---|---|---|---|---|---|
| | Was allocation adequate?* | Was an adequate method of randomization described | Were groups similar at the start of the study | the patients/caregivers blinded to the intervention | was the outcome ascertained blindly | What percent was lost to follow-up | Were all patient analyzed in the group to which they were assigned (intention-to-treat analysis)? |
| Assmus B,2010 | Y | Y | Y | Y | Y | 1.5 | N |
| Cao F,2009 | Y | Y | Y | NR | Y | 0 | Y |
| Colombo A,2011 | Y | Y | Y | Y | Y | 0 | Y |
| Castellani M,2010 | N | N | Y | N | N | 0 | N |
| Chen SL,2004 | Y | N | Y | Y | Y | 0 | Y |
| Gao LR,2013 | Y | Y | Y | N | Y | 4.7 | N |
| Ge J(2006) | Y | Y | Y | N | Y | 0 | y |
| Grajek S(2010) | Y | Y | Y | N | Y | 0 | N |
| Huikuri HV(2008) | Y | Y | Y | Y | Y | 3.7 | Y |
| Herbots L(2009) | Y | Y | Y | Y | Y | 1 | Y |
| Hirsch A (2011) | Y | Y | Y | N | Y | 2 | Y |
| Huang RC(2006) | Na | Na | Na | Na | Na | Na | Na |
| Janssens S(2006) | Y | Y | Y | Y | Y | 10 | Y |
| Lee JW(2014) | NR | N | Y | N | Y | 27.5 | N |
| Meluzín J(2008) | Nr | N | Y | N | Y | 0 | Y |
| Meyer GP(2006) | Y | Y | Y | Y | Y | 0 | Y |
| Nogueira FB(2009) | Y | Y | Y | N | Y | 0 | Y |
| Piepoli MF(2010) | Y | Y | Y | NR | NR | 0 | Y |
| Plewka M(2011) | N | N | Y | N | N | 13.3 | N |
| Penicka M(2007) | Y | Y | NR | NR | NR | 11 | N |
| Quyyumi AA (2011) | Y | N | Y | N | Y | 3.2 | N |
| Sürder D (2013) | Y | Y | Y | N | Y | 16.5 | N |
| Suárezde Lezo J (2007) | Y | Y | Y | NR | Y | 0 | Y |
| Tendera M (2009) | Y | Y | Y | N | Y | 0 | Y |
| Traverse JH (2012) | Y | Y | Y | Y | Y | 6.7 | N |
| Traverse JH (2011) | Y | N | Y | N | Y | 1.1 | Y |
| Traverse JH (2010) | Y | Y | Y | Y | Y | 0 | Y |
| Turan RG (2012) | NR | N | Y | N | Y | 0 | Y |
| Wen R (2005) | Y | N | Y | Y | Y | 0 | Y |
| Wöhrle J (2013) | Y | Y | Y | Y | Y | 4.7 | Y |
| Yao K (2009) | Y | Y | Y | NR | Y | 13 | N |

NA indicates not available; NR, not reported.

*Adequate means the use of central site, numeric code, opaque envelopes, drugs prepared by pharmacy, and other appropriate procedures as described by Juni et al.[50]

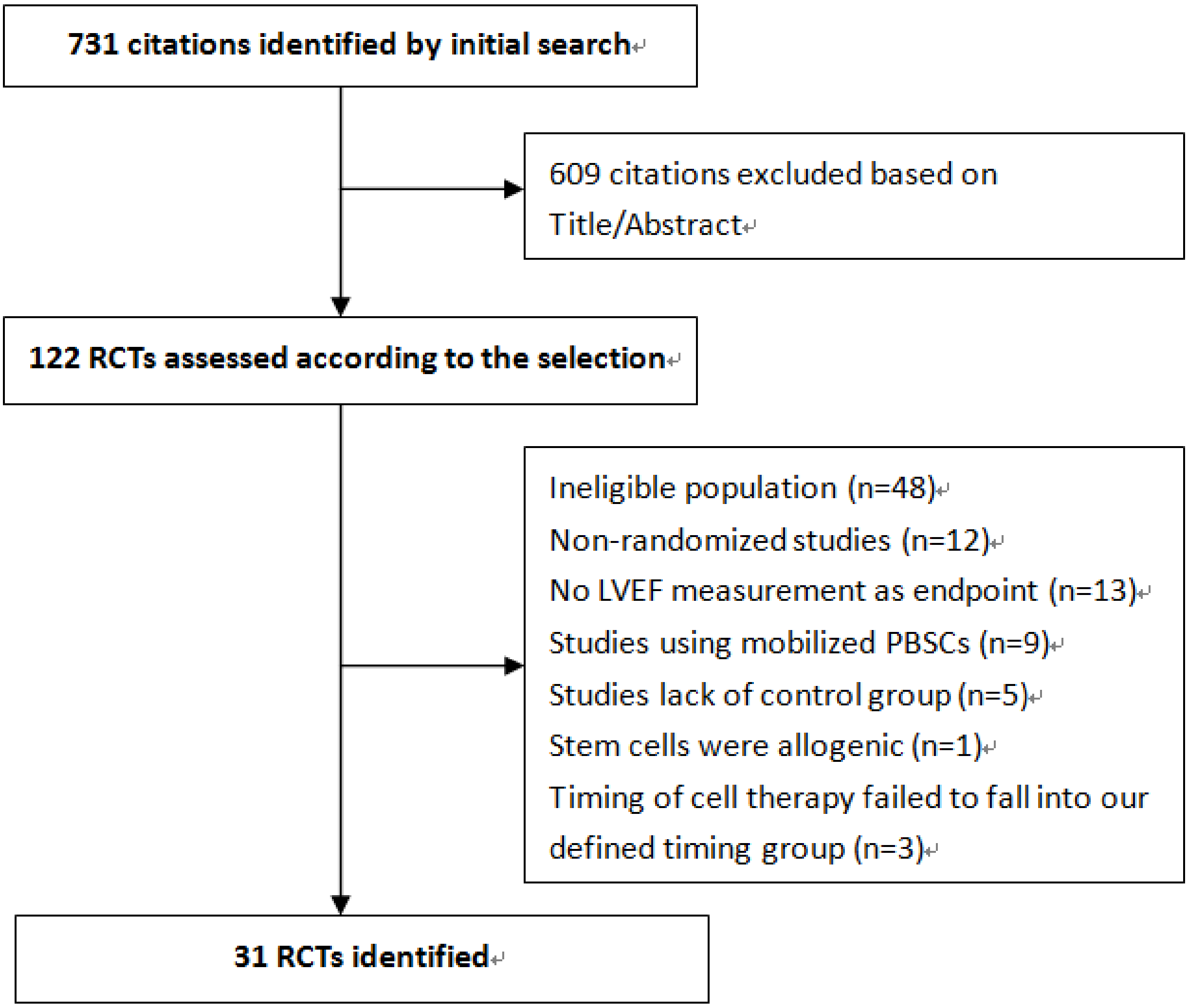


**Supplementary Figure S1. Flow Diagram of Eligible Studies of Bone Marrow Stem Cells Therapy in Patients with Acute Myocardial Infarction**. RCTs, randomized clinical trials. LVEF, left ventricular ejection fraction.

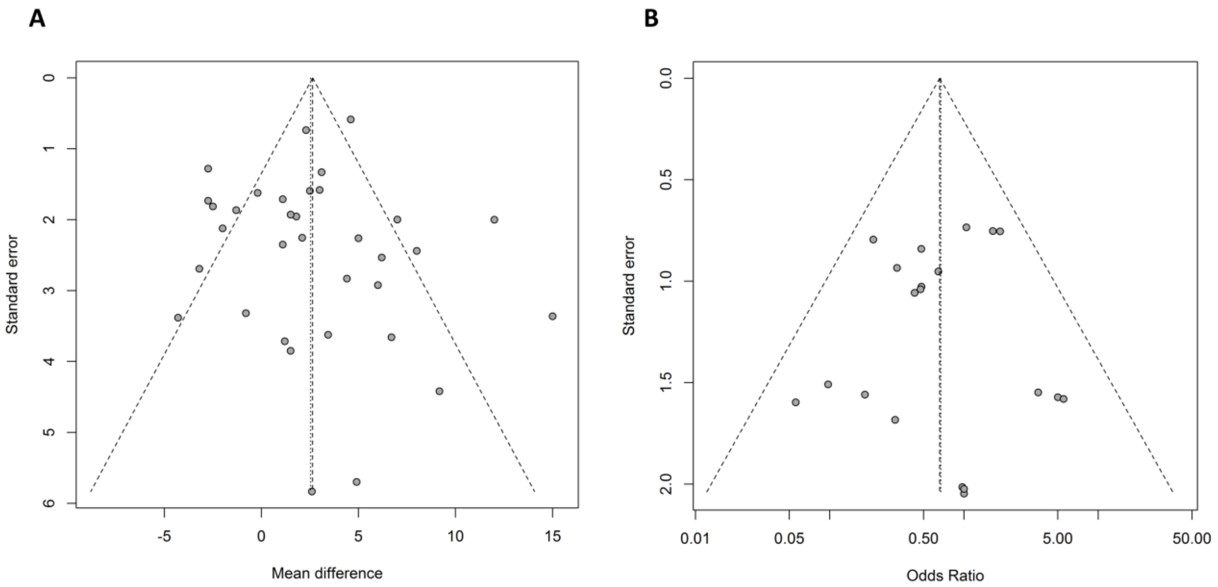


**Supplementary Figure S2. Funnel Plot. (A) Funnel Plot of Left Ventricular Ejection Friction, and (B) Funnel Plot of Major Adverse Cardiac Events**
